# Developing technology-based interventions for infectious diseases: ethical considerations for young sexual and gender minority people

**DOI:** 10.3389/frph.2023.1303218

**Published:** 2023-12-18

**Authors:** Cory J. Cascalheira, Tyler H. Pugh, Chenglin Hong, Michelle Birkett, Kathryn Macapagal, Ian W. Holloway

**Affiliations:** ^1^ Department of Counseling and Educational Psychology, New Mexico State University, Las Cruces, NM, United States; ^2^ Department of Social Policy and Intervention, University of Oxford, Oxford, United Kingdom; ^3^ Luskin School of Public Affairs, University of California, Los Angeles, Los Angeles, CA, United States; ^4^ Department of Medical Social Sciences, Feinberg School of Medicine, Northwestern University, Chicago, IL, United States; ^5^ Institute for Sexual and Gender Minority Health and Wellbeing, Northwestern University, Chicago, IL, United States

**Keywords:** infectious disease, LGBTQ+, young adults, emerging infections, adolescents, mobile health, technology-delivered interventions, eHealth

## Abstract

Compared to their heterosexual and cisgender peers, young sexual and gender minority (YSGM) people are more likely to contract sexually transmitted infections (STIs; e.g., HIV) and to face adverse consequences of emerging infections, such as COVID-19 and mpox. To reduce these sexual health disparities, technology-based interventions (TBIs) for STIs and emerging infections among YSGM adolescents and young adults have been developed. In this Perspective, we discuss ethical issues, ethical principles, and recommendations in the development and implementation of TBIs to address STIs and emerging infections among YSGM. Our discussion covers: (1) *confidentiality, privacy, and data security* (e.g., if TBI use is revealed, YSGM are at increased risk of discrimination and family rejection); (2) *empowerment and autonomy* (e.g., designing TBIs that can still function if YSGM users opt-out of multiple features and data collection requests); (3) *evidence-based and quality controlled* (e.g., going above and beyond minimum FDA effectiveness standards to protect vulnerable YSGM people); (4) *cultural sensitivity and tailoring* (e.g., using YSGM-specific models of prevention and intervention); (5) *balancing inclusivity vs. group specificity* (e.g., honoring YSGM heterogeneity); (6) *duty to care* (e.g., providing avenues to contact affirming healthcare professionals); (7) *equitable access* (e.g., prioritizing YSGM people living in low-resource, high-stigma areas); and (8) *digital temperance* (e.g., being careful with gamification because YSGM experience substantial screen time compared to their peers). We conclude that a community-engaged, YSGM-centered approach to TBI development and implementation is paramount to ethically preventing and treating STIs and emerging infections with innovative technology.

## 1. Sexually transmitted infections, emerging infections, and digital health interventions

Adolescence and young adulthood (12–25 years old) are developmental periods when sexual health disparities in sexually transmitted infections [STIs; e.g., chlamydia, gonorrhea, human immunodeficiency virus (HIV)] emerge between young sexual and gender minority (YSGM) people (1) and their heterosexual and cisgender counterparts (2, 3). Without intervention, STI disparities persist across the life course (4). Evidence also indicates that YSGM people are at greater likelihood of (1) contracting certain emerging infections [e.g., mpox; (5)] and (2) developing more severe health outcomes from emerging infections [e.g., COVID-19; (6, 7)]. As such, there is an urgent need to develop and implement innovative interventions to reduce these sexual health disparities in YSGM people (8).

Technology-based interventions (TBIs), or the use of mobile and internet technologies for health prevention and remediation, are innovative solutions to reducing disparities in STIs and emerging infections among YSGM people. TBIs for STIs typically focus on enhancing STI prevention messaging, increasing STI testing, bolstering pre-exposure prophylaxis (PrEP) uptake, surveilling STI prevalence, improving adherence to medication, and connecting to wraparound services (9). Evidence indicates that TBIs for STIs can significantly improve medication adherence (e.g., pre-exposure prophylaxis) and clinic attendance, significantly reduce transmission risk behaviors, and are perceived as acceptable and feasible (1, 10). Among YSGM people, many of whom have limited knowledge of STIs given their age and reduced access to familial or peer supports, TBIs have the added benefit of accessibility outside of discriminatory medical environments (11). However, substantial gaps in TBIs for STIs and emerging infections remain, such as developing TBIs for subgroups of the YSGM community [e.g., sexual minority female adolescents (1)] and developing TBIs for emerging infections. As momentum builds to develop and implement TBIs to address these gaps, stakeholders must consider pertinent ethical issues and principles in working with YSGM people. In this Perspective, we discuss these ethical issues and principles as well as provide recommendations to navigate them.

## 2. Ethical issues and recommendations for intervening with YSGM people

Drawing from the ethical frameworks of the Belmont Report (12) and the American Psychological Association code of ethics (13), we identify eight ethical issues and principles that raise important questions during TBI development and implementation for YSGM at risk for STIs and emerging infections. Table 1 summarizes this Perspective's central recommendations as questions.

**Table 1:** A summary of ethical considerations.

Ethical issue/principle	Questions to consider
Confidentiality, privacy, and data security	• How have I considered how third parties and other external entities might cause harm if access to TBI data is acquired? • If working with a YSGM person below age of majority, have I considered the possibility of familial rejection and/or bullying if I include them in the study? What protections have I included to mitigate these risks? • Have I evaluated TBI processes and content for materials that might unintentionally disclose someone's YSGM status? • To what extent have I followed best practices in confidentiality, privacy, and data security? • What additional special considerations, if any, are required for underage YSGM people? • Do I know the local laws governing informed consent in my jurisdiction? • If my TBI uses artificial intelligence, have I considered the unintended consequences of publishing the models, features, and data? • Have I attempted to include YSGM people and other community leaders in the TBI development and implementation process? • Have I done my due diligence in informing YSGM of the limitations of data security and the potential risks involved in the use of the TBI?
Empowerment and autonomy	• Am I highlighting and uplifting YSGM voices at every step of the intervention process? How am I ensuring that those voices are not overlooked? • Have I provided appropriate information of consent to both the parent or guardian and YSGM participants before beginning? • Am I evaluating TBI content for indicators of manipulative or ambiguous language? • How might I include personalized options within the TBI for YSGM to tailor the intervention to their needs? • Are there clear and numerous options to withdraw throughout the TBI? • Have I involved members of the local and/or LGBTQ+ community to ensure language is empowering and respectful?
Evidence-based and quality controlled	• Am I rooting my research/TBI in previous high-quality evidence-based literature from the field? • Have I constructed a clear logic model for my TBI? • Have I accurately identified how I will measure for and assess the quality of the TBI to ensure reliability? • Have I explored ways to validate the effectiveness of the intervention in improving health outcomes? Have I established a pipeline for the validation methods to be documented and published? • Am I engaging with health professionals to ensure the TBI is upholding the rigor of an evidence-based intervention?
Cultural sensitivity and tailoring	• What intersectional identities pertinent to my research are included in the study? • Have I and other investigators drafted a statement of reflexivity to make transparent and minimize bias? • Have I incorporated perspectives of the target population in which the TBI is utilized to ensure relevant co-creation? • Have I explored cultural differences that may affect the language use or delivery of the TBI? • Am I utilizing the voices of different backgrounds to ensure inclusive, tailored language where necessary? • Can YSGM receiving the TBI have access to LGBTQ-identifying clinicians or peer advocates?
Inclusivity vs. group specificity	• Have I adequately engaged with the community to understand which subgroups most need the intervention? • Have I sufficiently consulted with a YSGM sample that reflects likely end-users to ensure needs from across the community are met? • Am I addressing intersectionality within YSGM interventions? Is my TBI only serving white, affluent, or cisgender YSGM? • Can I provide a direction toward alternative resources for those who aren't directly served by the TBI?
Duty to care	• Am I leading with empathy (e.g., active listening, using appropriate pronouns and identity-affirming language) when interacting with YSGM? • Am I upholding the ethical principles of medicine in caring for YSGM? • Is everyone who is directly handling the TBI briefed on responsibilities and obligations to providing service for YSGM in need? • Have I acknowledged the limitations of the TBI in providing care and compiled resources to redirect YSGM with needs beyond its capacity? • Have I incorporated ongoing assessments of care to ensure harm is not inflicted in implementation of the TBI, indirectly or otherwise? • Do I have a plan for regular technological and content updates?
Equitable access	• Have I done my best in ensuring all TBIs are easy to access, regardless of technological access or background? • How have I provided accessible alternatives to the main-line form of delivery (closed captions, screen reading, alt texts, etc.)? • Have I translated the TBI into relevant languages dependent on target population? • Can I ensure that closeted YSGM are not outed in the process of accessing the TBI? • How have I designed a distribution plan for the TBI to ensure all those who could be served are able to utilize it? • How have I devised a roll-out plan for YSGM who are located in rural regions or geographies with anti-LGBTQ+ policies?
Digital temperance	• To what extent have we implemented sufficient safeguards to prevent digital addiction? • How have we incorporated activities outside of digital means to minimize screen time and encourage non-virtual experiences? • Has the TBI been healthily incremented to balance digital exposure? • Do I have measures of individual usage hours to monitor engagement with the TBI? • Do I have means of addressing signs of addictive tendencies with the YSGM or guardian?

### 2.1. Confidentiality, privacy, and data security

Maintaining confidentiality, privacy, and data security is paramount for TBI research, but additional care is required when working with YSGM people because the consequences of data breach can be dire. Adolescence and young adulthood are periods when many YSGM people develop their identities and decide if and how to disclose their YSGM status (14). Identity disclosure during research has important interpersonal implications (15), and thus, researchers and clinicians must consider whether third parties might cause harm if access to TBI data is acquired (16). For example, some families may reject their YSGM teenager upon disclosure of their SGM status, possibly resulting in homelessness (17). Identity disclosure can also increase the risk of bullying and victimization in high-stigma areas (18). Unintentional disclosure might occur during TBI development and validation [e.g., having one YSGM person reach out to a closeted YSGM person via snowball sampling; (19)], content design (e.g., using rainbow images in an app's logo design), content storage [e.g., content stored on the cloud vs. locally on a YSGM person's smartphone; (20, 21)], or implementation (e.g., providing the TBI at a clinic where the YSGM person is out to the provider but not the parent).

In addition to best practices in maximizing confidentiality, privacy, and data security [e.g., anonymity, encryption, data held separately from identifiers; see (22)], we recommend the following.

1. Decide on the age range of the intended end user (23), as some issues related to unintentional identity disclosure can be avoided with older YSGM people.
2. Know the local laws about consent. In the United States, for example, parental or guardian permission may be waived if sufficient safeguards are available to protect children or adolescents, and provided that the waiver does not contradict laws (24).
3. In TBIs that use artificial intelligence, ensure that reverse engineering of features and models is unlikely by restricting the access and use of preprocessed data.
4. Conduct focus groups to understand how YSGM people might want the TBI presented (e.g., what images and language to use) to maximize confidentiality and privacy (25).
5. Involve YSGM at all stages of TBI development and implementation (e.g., advisory boards to provide input on local needs, technology preferences, concept design, recruitment materials, distribution points; stipends for youth to design social media outreach and visualizations of study findings) to ensure the processes are embedded in community norms and prioritize digital rights.

### 2.2. Empowerment and autonomy

Non-affirming parenting practices (26), peer bullying (27), anti-YSGM language in public spaces (28), and anti-YSGM laws (29) disempower YSGM people. Because disempowering experiences drive YSGM health disparities (30), it is important to center empowerment and autonomy in TBIs. One method is to design TBIs that adequately function if YSGM users opt-out of multiple features and data collection requests. TBI developers could also provide opportunities to promote autonomous decision-making by designing modular TBIs [vs. linear and constrained; (31)], using visually based and easy to comprehend informed consent materials, and eliciting informed consent throughout the TBI (23). Another method might be featuring YSGM people as co-authors or co-developers of the TBI. For instance, if focus groups or advisory boards are used throughout TBI development and implementation as recommended in this perspective, YSGM who participate in such activities could be featured somewhere on the TBI explaining their contributions. This representation could convey a message of “for us by us” which, in turn, could help other YSGM people feel seen and valued.

### 2.3. Evidence-based and quality controlled

In the United States, most TBIs are classified as low-to-moderate risk by the Food and Drug Administration (i.e., the nation's regulatory body for healthcare products), so they receive minimal premarket clinical testing (32). In this regulatory environment, venture capitalists are investing heavily in TBIs [$637 million in 2019; (33)]. As a result, TBIs may enter the market without adequate effectiveness evidence, leaving the burden of determining effectiveness on individual providers (16). Furthermore, the regulation of artificial intelligence in healthcare, including TBIs, is a moving target (34). These regulatory realities raise concerns about the extent to which TBIs are based on scientific evidence.

Regulatory limitations also underscore the potential for harm in rapid TBI development and implementation. YSGM people, given their increased vulnerability in healthcare settings relative to heterosexual and cisgender people (35), require extra care to reduce the potential of “technology-facilitated abuse” (36)—that is, the harm that derives from TBIs with poor validity, accuracy, safety, and scientific rigor. Even if a local jurisdiction does not require extensive effectiveness evidence, we charge TBI developers and providers to implement TBIs with YSGM people if and only if: (a) the TBI is technologically stable, has high fidelity, and yields clinically meaningful results (37); (b) implementation stakeholders are educated on the scientific evidence behind the TBI; (c) the TBI has a robust monitoring and evaluation plan (38); and (d) the TBI presents accurate claims of its effectiveness for YSGM people.

### 2.4. Cultural sensitivity and tailoring

Without cultural sensitivity and tailoring, TBI uptake and effectiveness among YSGM people may be suboptimal. Research suggests that culturally tailored interventions tend to be effective among YSGM people (39–41), especially for non-White YSGM (42) and for outcomes like HIV (43, 44). From an implementation perspective, clinical practitioners who lack understanding of YSGM issues and concerns (45) may hinder TBI uptake. Thus, we caution against adapting extant TBIs to YSGM people without adequate consideration of how intervention components are delivered in a YSGM-affirming manner. Instead:

• Involve YSGM people at all stages of TBI development and implementation to reduce the chances that a TBI perpetuates stereotypes, promotes discrimination, or causes unintended harm;
• Co-design TBIs with clinical providers and end users (46–48) to help increase the health literacy of YSGM people and the cultural literacy of clinical providers.
• Use a community-engaged, YSGM-centered approach to TBI development and implementation to create partnerships with local YSGM-affirming clinical providers, local YSGM community organizations, national advocacy organizations, and academic research centers.

Special consideration for TBIs enhanced by artificial intelligence [e.g., a chatbot to increase demand for STI medications; (49)] is warranted because anti-YSGM bias may be integrated into the technological architecture of specific models [e.g., large language models (50)]. In these special cases, we recommend regular algorithmic auditing and validation [for more information, see (51)] as well as the creation of *community-tailored algorithms*, or artificial intelligence algorithms trained from the ground up using data from YSGM people.

### 2.5. Inclusivity vs. group specificity

Rates of STIs differ by gender identity and sexual orientation (52), necessitating the development of differentiated interventions for YSGM people. Moreover, majority group researchers, clinical providers, or public health officials may treat YSGM communities as a monolith even though members have intersectional identities (53), unique constellations of stressful experiences (54), and diverse healthcare experiences (55). Because YSGM people are a historically neglected population (56, 57), it can be tempting to overlook the heterogeneity of this group when developing TBIs for STIs and emerging infections. We encourage the careful consideration of whether the mechanisms of the TBI are broadly applicable to YSGM people, the execution of thorough acceptability and feasibility studies with YSGM subgroups, the matching of YSGM people with subgroup-specific interventions (58), and the adaptation of existing TBIs to specific subgroups [e.g., transgender youth; (1)].

### 2.6. Duty to care

TBIs should prioritize the long-term well-being of YSGM people with sustainable plans for TBI maintenance and appropriate connections to other forms of care. Developing, releasing, and forgetting a TBI is unacceptable because YSGM people constitute a vulnerable population. For instance, with an infection like HIV, it could be useful to design TBIs to connect to wraparound services. Furthermore, YSGM people may perceive TBIs for STIs and emergent infections as spaces to seek help for comorbid concerns, especially since YSGM-specific TBIs for mental health conditions are relatively lacking (1). Because suicide is alarmingly high in this population (59), careful attention to how high-risk suicidal behaviors may be disclosed in interactive components (e.g., forums, web chat) is warranted (60, 61). We recommend creating TBIs that have clear pathways to affirming healthcare providers, easy to access crisis lines, and ongoing plans for monitoring risk if interactive components are featured (e.g., passive-sensing suicide risk detection systems that alert clinical team members to perform a suicide assessment). For emergent infections, we encourage TBI developers to design technologies that connect to other forms of care (e.g., another TBI for sexual health) in the event that an infection is rapidly controlled.

We also recommend regular technological and content updates. Technological updates are important to ensure the TBI syncs with operating systems and does not pose a safety risk. Larger technological updates may be required if the TBI technology becomes substantially outdated or no longer meets user expectations. Regular content updates are also crucial. Information on STIs and emerging infections can rapidly develop, so it is important to have content that reflects new recommendations and science.

### 2.7. Equitable access

YSGM people who might most benefit from TBIs for STIs or emergent infections (i.e., YSGM people in high-stigma, low-resource areas) are also least likely to have the TBI developed with them. Many TBIs for YSGM people are developed in urban centers where YSGM people cluster (1, 44). Even in urban areas, facilitators of and barriers to healthcare resources within the YSGM community is not uniform (62). Thus, although it may be practical to roll out a TBI in urban centers with YSGM people that are easier to recruit (e.g., gay men presenting regularly at a sexual health clinic), we recommend that TBI stakeholders have a plan to scale up distribution of the TBI to YSGM people in high-need, underserved areas. Moreover, through the inequity of the technological landscape, access to broadband, Wi-Fi, and other technological pre-requisites to receiving care are inevitably variable. In response, adaptable interventions that can remain versatile to the access barriers of a given region become essential.

### 2.8. Digital temperance

While TBIs offer solutions to address the disparities in STIs and emerging infectious disease outbreaks, it is important to acknowledge and minimize the potential harm in their implementation. Many TBIs inevitably increase the screen time among users. Previous research suggests that increased screen time is associated with poorer mental health, increased behavioral problems, worsening sleep quality, and other outcomes including poor academic performance (63, 64). Therefore, TBI development and implementation must consider the potential downside of excessive technology use. Similarly, there is a growing trend of integrating gamification and serious gaming in health and wellbeing promotion in recent years (65, 66). While these approaches could generate positive behavioral changes and cognitive outcomes, excessive use of such elements could lead to addiction-like behaviors, contributing to the already existing mental health inequities among YSGM. These considerations are particularly important given the recent statement from the U.S. Surgeon General on social media use and its impact on adolescents' mental health (67). Therefore, it is crucial for TBI developers and clinicians to strike a balance between optimizing TBI engagement for positive behavior changes and maintaining a moderate amount of technology use.

## 3. Discussion

This Perspective discussed eight ethical issues and principles is designing and deploying TBIs for STIs and emerging infections, focusing on YSGM people as the target population. Ultimately, a community-engaged, YSGM-centered approach to TBI development and implementation is paramount to ethically treating STIs and emerging infections with innovative technology. Although some of the ethical issues, principles, and recommendations in this Perspective cut across populations, attention to how ethical considerations apply to YSGM people is crucial given the ethical infractions of extant computational technologies with this population (68, 69) as well as the health disparities observed between YSGM people and their heterosexual and cisgender peers (2, 3). Ethically robust TBI development and implementation is not only sound science, but is also a way to ensure systems of personal and public health remain spaces of help, healing, and hope at a time when YSGM people face an unprecedented assault on their basic human rights (29).

## Data Availability

The original contributions presented in the study are included in the article/Supplementary Material, further inquiries can be directed to the corresponding author.
